# Optimization for peptide sample preparation for urine peptidomics

**DOI:** 10.1186/1559-0275-11-7

**Published:** 2014-02-25

**Authors:** Tara K Sigdel, Carrie D Nicora, Szu-Chuan Hsieh, Hong Dai, Wei-Jun Qian, David G Camp, Minnie M Sarwal

**Affiliations:** 1 California Pacific Medical Center Research Institute, 475 Brannan St., Ste 220, San Francisco, CA 9410, USA; 2 Biological Sciences Division, Pacific Northwest National Laboratory, Richland, WA 99352, USA

**Keywords:** Urine, Biomarker, Peptidomics, Biomarker discovery, Proteomics, Transplantation

## Abstract

Analysis of native or endogenous peptides in biofluids can provide valuable insights into disease mechanisms. Furthermore, the detected peptides may also have utility as potential biomarkers for non-invasive monitoring of human diseases. The non-invasive nature of urine collection and the abundance of peptides in the urine makes analysis by high-throughput ‘peptidomics’ methods , an attractive approach for investigating the pathogenesis of renal disease. However, urine peptidomics methodologies can be problematic with regards to difficulties associated with sample preparation. The urine matrix can provide significant background interference in making the analytical measurements that it hampers both the identification of peptides and the depth of the peptidomics read when utilizing LC-MS based peptidome analysis. We report on a novel adaptation of the standard solid phase extraction (SPE) method to a modified SPE (mSPE) approach for improved peptide yield and analysis sensitivity with LC-MS based peptidomics in terms of time, cost, clogging of the LC-MS column, peptide yield, peptide quality, and number of peptides identified by each method. Expense and time requirements were comparable for both SPE and mSPE, but more interfering contaminants from the urine matrix were evident in the SPE preparations (e.g., clogging of the LC-MS columns, yellowish background coloration of prepared samples due to retained urobilin, lower peptide yields) when compared to the mSPE method. When we compared data from technical replicates of 4 runs, the mSPE method provided significantly improved efficiencies for the preparation of samples from urine (e.g., mSPE peptide identification 82% versus 18% with SPE; p = 8.92E-05). Additionally, peptide identifications, when applying the mSPE method, highlighted the biology of differential activation of urine peptidases during acute renal transplant rejection with distinct laddering of specific peptides, which was obscured for most proteins when utilizing the conventional SPE method. In conclusion, the mSPE method was found to be superior to the conventional, standard SPE method for urine peptide sample preparation when applying LC-MS peptidomics analysis due to the optimized sample clean up that provided improved experimental inference from the confidently identified peptides.

## Background

Disease specific biomarkers remain as an unmet need in an overwhelming majority of cases for monitoring human health. Though there is an ever increasing effort to identify specific and sensitive biomarkers for monitoring human health and for the early detection of disease onset, many hurdles still exist in identifying effective biomarkers [1]. In this context, urine could prove to be an important proximal fluid in providing biomarkers that could be tested noninvasively [2]. The choice of urine is particularly useful in the context of kidney and urinary system related diseases such as acute and chronic kidney diseases, diseases with either the urinary bladder or prostrate, and kidney transplantation [2-9]. Circulating peptides in urine are to be considered potentially useful as biomarkers as they are both abundant and easily accessible. Increased efforts to identify and analyze these valuable peptides has been undertaken as they are thought to contribute insights into disease mechanisms as well as providing potential biomarkers for diagnosis, prognosis, therapeutic intervention and treatment outcome [10]. The emergence of new and sophisticated methods of molecular profiling have aided in our ability to analyze peptides in complex biological mixtures in combination with the analysis of their degradation patterns. This information may provide important clues about underlying (patho) physiological processes [11,12]. Different technology platforms have been applied for the study of urine proteins and peptides such as LC-ESI-MS [13], LC-MALDI-MS [14], CE-MS [15], and SELDI-TOF [16]. While SELDI-TOF and CE-MS are relatively easy platforms to operate their weakness is that they do not allow for peptide identification resulting in incomplete biomarker discovery and identification. Our previous report presented our findings that applied a LC-MALDI-MS approach where a standard solid phase extraction (SPE) method for peptide enrichment and purification was utilized [14]. Unlike urine peptide analysis by MALDI [17-19] urine peptidomics analysis by LC-MS is highly sensitive to the presence of unidentified contaminants that exist in urine, which if not effectively removed by further purification, impair the performance of the LC-MS analysis. When the standard solid phase extraction (SPE) [20] method of urine peptide extraction and purification was utilized for sample preparation, persisting contaminants in the urine, inclusive of urobilin and urobilinogen, clog the LC column and interfere with the assay performance (Sigdel et al., unpublished data) Herein we report a mandatory modification of the SPE method, termed modified-SPE (mSPE) method, which provides optimal peptide extraction and purification for urinary peptide analysis by LC-MS.

## Results and discussions

### Comparison of urine peptide extraction by SPE and mSPE methods

**Figure 1:**
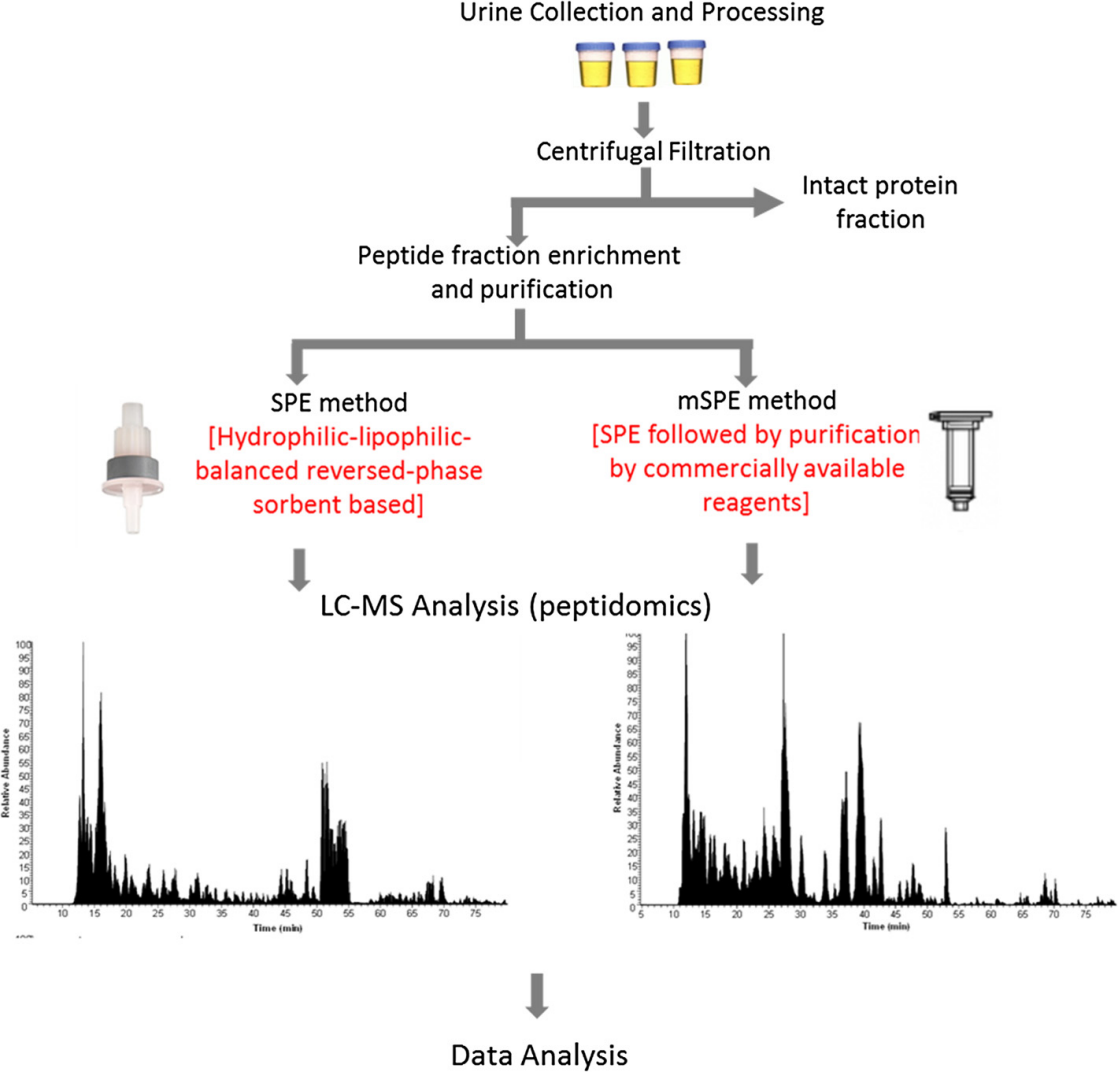
**Schematics of peptide extraction and purification strategy Urine samples were passed through a centrifugal filtration column to separate intact (larger MW proteins) from native peptides (smaller MW peptides).** The peptide fraction was subjected to solid phase extraction (SPE) and modified SPE (mSPE) methods described in the Methods section. The resulting peptides were analyzed by LC-MS and identified for comparison of robustness of the two isolation and purification methods.

**Figure 2:**
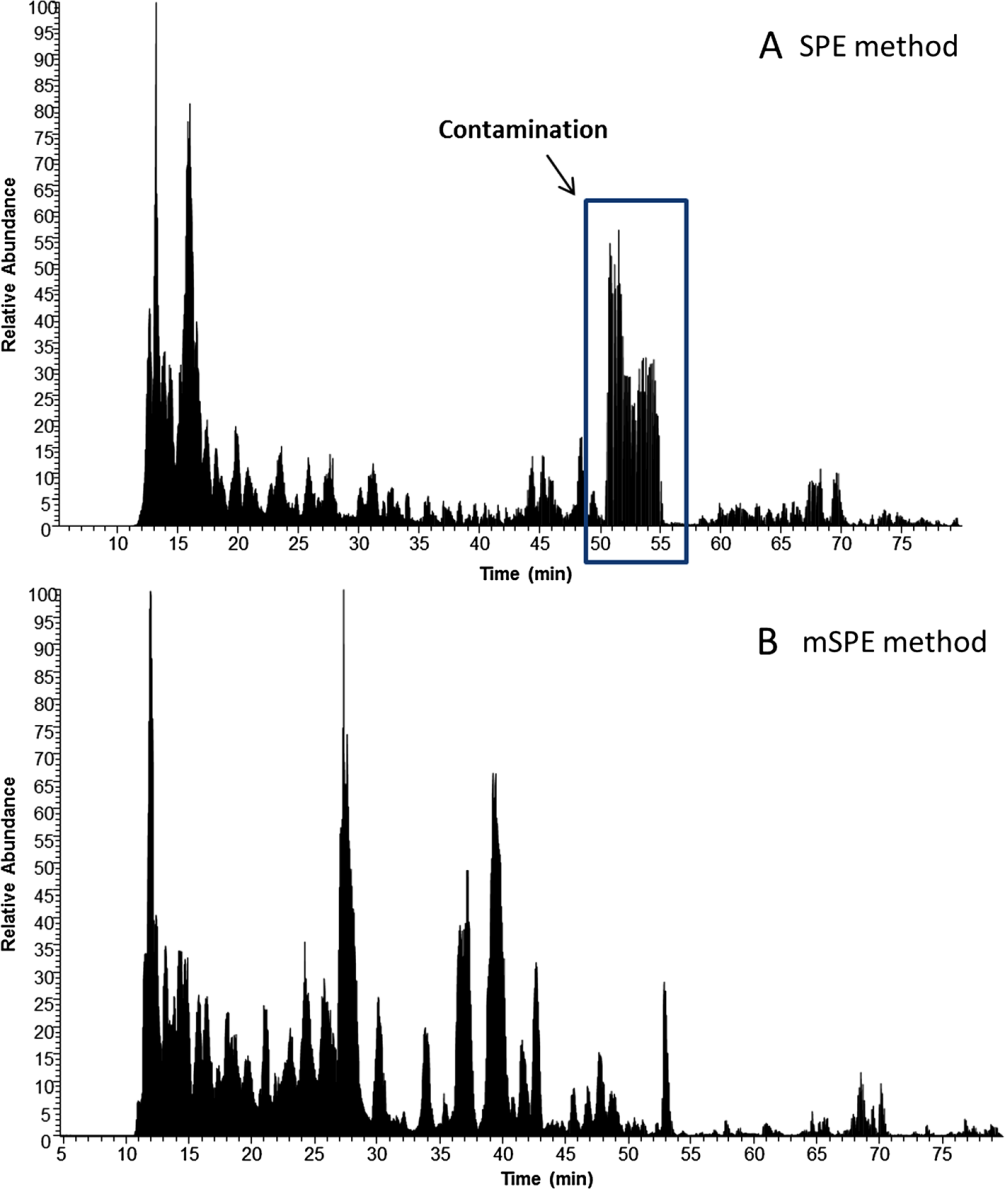
**Comparison of representative chromatograms for SPE and mSPE. (A)** The LC chromatogram from the SPE method contains a large region of low intensity peaks presumably due to the ionization suppression effect from the contaminating species. **(B)** The LC chromatogram from the mSPE method displays well-distributed peaks throughout the LC span.

The study compared two peptide isolation methods to isolate urine peptides for peptidome analysis by LC-MS. The first method used hydrophilic-lipophilic-balanced reversed-phase sorbent-based (a mixture of two monomers, hydrophilic N-vinylpyrrolidone and lipophilic divinylbenzene) solid phase extraction (SPE) method. The second method is called modified SPE (mSPE). In this second method, we used peptides isolated from SPE and subjected them to a second step of purification using a processed silicon carbide resin in a pH dependent manner. The activation of the resin and peptide binding involved a low pH buffer (pH 3.5-4) and elution is performed with a Na-phosphate buffer (pH 12.5). The comparison of the two methods, SPE versus mSPE, is summarized in Table 1 and Figure 1. Both methods were able to extract sufficient urine peptides for LC-MS analysis. The time required by each of the two methods to isolate peptides from urine was comparable (6 h for SPE vs 8 h for mSPE); as was the cost of supplies ($12 for SPE and $15 for mSPE). Starting with a 10 mL volume of pooled urine, the SPE method yielded a substantially larger pellet when compared to the mSPE method, and the crude analysis of the peptide content of the SPE pellet initially suggested that the method had a greater peptide yield (13.6 μg by SPE compared to 1.2 μg by the mSPE method). It was determined that the SPE peptide extract contained additional contaminating pigments and other unidentified matter that gave the SPE pellet a yellowish color that was both murky and opaque. We tested for any influence of the colored contaminant on BCA assay by assaying the washed colored component by BCA assay. We found out that the contaminant in the wash solution had no protein and peptide in it indicating there was no protein-like substance in it. This confirmed that there was no influence of this colored substance on BCA assay. Additionally, the SPE peptide extract could not be processed for subsequent LC-MS analysis as the associated contaminants clogged the capillary LC columns after only 1–3 sample injections followed by LC gradient separations through the analytical column. Sample processing by the mSPE method, resulted in a peptide extract that had a clear and colorless appearance and, importantly, did not clog the LC columns, irrespective of the number of sample injections and LC separations, indicating that contaminants present in the SOE-prepared urine had been effectively removed by the mSPE method. Notably, there was a striking difference in the peptide quality resulting from each method. Two liquid chromatograms that compare the peptide quality resulting from both methods are displayed in Figure 2. The LC chromatogram from the mSPE method shows well-distributed peaks throughout the LC span while the chromatogram from the SPE method contains a large region of low intensity peaks presumably due to the ionization suppression effect from the contamination species.

**Table 1:** A comparison of SPE and mSPE methods for urine peptide extraction

	**SPE method**	**mSPE method**
**Sample processing time**	6 h	8 h
**Sample processing cost**	$12	$15
**Peptide quantity as measured by BCA assay**	13.6 μg	1.2 μg
**Sample Appearance and Impact on LC column**	Murky and clogs LC column	Clear and no clogging of LC column
**% peptides identified ompared to the total identified peptides**	18%	82%

### Improved urine peptidome data quality by mSPE modification

**Figure 3:**
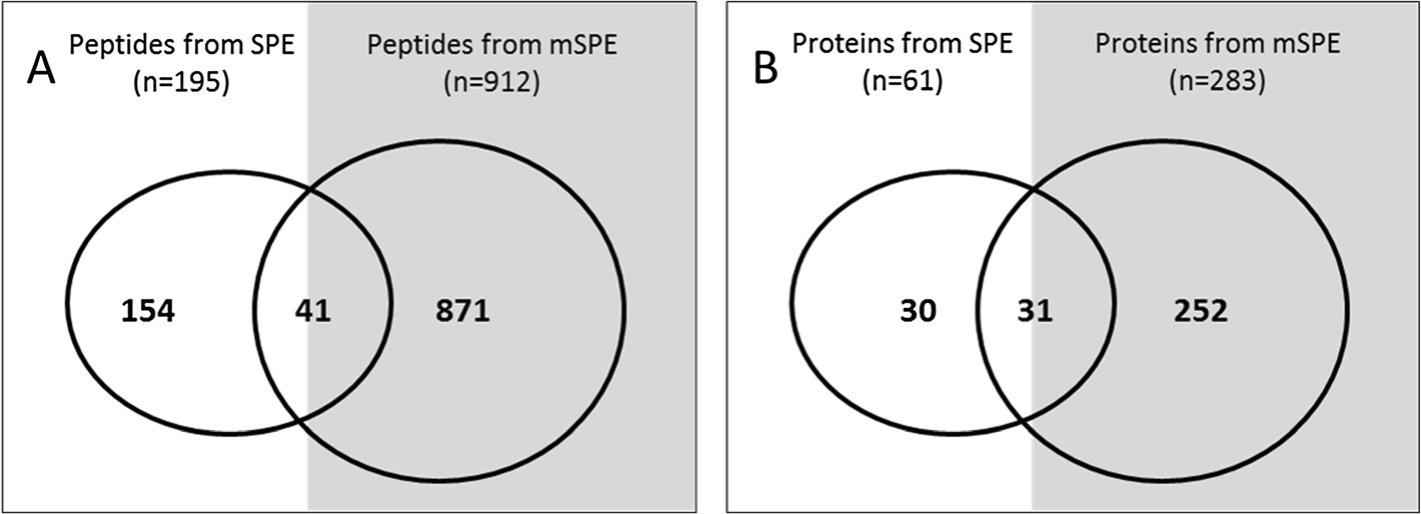
**Comparison of peptide and protein identifications between peptides extracted by either solid phase extraction (SPE) or modified SPE (mSPE) methods.** A total of 1066 unique urine peptides that originated from 305 unique human proteins were identified. Peptide extract from SPE and mSPE methods yielded **(A)** 195 and 912 unique peptides **(B)** from 61 and 283 unique proteins. Among the identified peptides 41 unique peptides from 31 unique proteins were commonly identified.

Using 4 technical replicates in each method, the mSPE method identified approximately 4.5 times more peptides than the SPE method; the mSPE method identified 912 unique peptides from 283 unique proteins, whereas the SPE method only identified 195 unique peptides from 61 unique proteins (Additional file 1: Figure S1). Between both methods, a total of 1066 unique urine peptides (only 41 unique peptides were overlapping between the two methods) were identified from 305 unique human proteins of which the SPE method only identified 18% of total identified peptides, whereas 82% of total identified peptides were picked up in samples processed by the mSPE method, which is a significant enhancement in peptide identification by the mSPE analysis method (p = 8.92E^-05^) (Figure 3) (Additional file 2).

Endogenous peptides have been profiled and studied in many health issues that include brain, kidney, and neurological diseases [14,17,19,21-23]. Of significance, analysis of the identified urine peptides in this study when applying either method highlighted novel biology in urine samples obtained from renal allograft patients with acute allograft rejection [14,24] (Table 2). However, the greater number of peptides identified by the mSPE method revealed a ladder-like pattern in the sequence of the identified peptides, suggesting selective activation of peptidases in urine; for example, the Alpha 1-antitrypsin peptide pattern shown in Table 2. Even though a similar cleavage pattern is observed for the peptides analyzed from SPE-processed samples, the pattern displays only an incomplete ladder. A list of the top 25 proteins based on their peptide abundances in urine and their corresponding identified peptides in each method is presented in Table 3.

**Table 2:** A ladder-like pattern was identified among the peptides identified by mSPE method

**Alpha 1-Antitrypsin**	**Identified in SPE**	**Identified in mSPE**
A.EDPQGDAAQKTDTSHHDQDHPTFNKITPNLAEFAFS.L	No	Yes
A.EDPQGDAAQKTDTSHHDQDHPTFNKITPNLAEFA.F	No	Yes
A.EDPQGDAAQKTDTSHHDQDHPTFNKITPNLAEF.A	No	Yes
A.EDPQGDAAQKTDTSHHDQDHPTFNKITPNLAE.F	Yes	Yes
A.EDPQGDAAQKTDTSHHDQDHPTFNKITPNLA.E	Yes	Yes
A.EDPQGDAAQKTDTSHHDQDHPTFNKITPNL.A	Yes	Yes
A.EDPQGDAAQKTDTSHHDQDHPTFNKITPN.L	Yes	Yes
A.EDPQGDAAQKTDTSHHDQDHPTFNKITP.N	No	Yes
A.EDPQGDAAQKTDTSHHDQDHPTFNKIT.P	No	Yes
A.EDPQGDAAQKTDTSHHDQDHPTFNK.I	Yes	Yes
A.EDPQGDAAQKTDTSHHDQDHPTFN.K	Yes	Yes
**Fibrinogen Alpha**		
A.DEAGSEADHEGTHSTKRGHAKSRPV.R	Yes	Yes
A.DEAGSEADHEGTHSTKRGHAKSRP.V	Yes	Yes
A.DEAGSEADHEGTHSTKRGHAKS.R	No	Yes
A.DEAGSEADHEGTHSTKRGHAK.S	No	Yes
A.DEAGSEADHEGTHSTKRGHA.K	Yes	Yes
A.DEAGSEADHEGTHSTKRG.H	Yes	Yes
A.DEAGSEADHEGTHSTKR.G	Yes	Yes
A.DEAGSEADHEGTHSTK.R	Yes	Yes
**Serum Albumin**		
R.DAHKSEVAHRFKDLGEENFKALV.L	No	Yes
R.DAHKSEVAHRFKDLGEENFKAL.V	No	Yes
R.DAHKSEVAHRFKDLGEENFKA.L	No	Yes
R.DAHKSEVAHRFKDLGEENFK.A	No	Yes
R.DAHKSEVAHRFKDLGEENF.K	No	Yes
R.DAHKSEVAHRFKDLGEEN.F	No	Yes
R.DAHKSEVAHRFKDLGEE.N	No	Yes
**Osteopontin**		
K.AIPVAQDLNAPSDWDSRGKDSYETSQLDDQSAETHSHKQSRL.Y	No	Yes
K.AIPVAQDLNAPSDWDSRGKDSYETSQLDDQSAETHSHKQSR.L	No	Yes
K.AIPVAQDLNAPSDWDSRGKDSYETSQLDDQSAETHSHK.Q	No	Yes
K.AIPVAQDLNAPSDWDSRGKDSYETSQLDDQSAETHSH.K	No	Yes

**Table 3:** Comparison on performance of peptide identification by SPE and mSPE methods

**Protein**	**Unique peptides from SPE**	**Unique peptides from mSPE**
ALBU	6	115
OSTP	3	106
A1AT	24	44
CO1A1	21	29
FIBA	24	19
ATNG	0	24
CD99	6	25
APOA4	0	25
CMGA	0	23
PIGR	11	16
VGF	5	20
TYB4	1	22
B2MG	1	22
APOA1	7	13
1C04	0	12
MOTI	0	11
GELS	1	11
SCG1	0	10
SRGN	0	12
HRG	1	10
LTBP4	0	9
THRB	0	10
UROM	10	1
FETUA	7	5
CALX	0	8

## Conclusion

In conclusion, analysis of urine peptides can provide valuable disease and renal injury specific biomarkers and provide additional insights into the underlying (patho) physical processes. The LC-MS approach represents a powerful platform for peptidome analysis and allows for significantly greater coverage of peptide identifications than other approaches (e.g., MALDI [ref]). This study demonstrated that exquisite attention needs to be paid to the methodology selection for the preparation of urine peptidome samples, in order to obtain the highest purity samples as possible, free from the inherent contaminants that exist in urine, such as urobilin, urobilinogen (likely contributing to the yellowish color of the prep by the SPE method), urea, creatinine, uric acid, carbohydrates, hormones, fatty acids, pigments, and inorganic ions. The novel modification of the SPE method followed by a second step that further purifies urine peptides using processed silicon carbide in a pH-dependent manner highlighted here allowed for preparation of a purified urine samples for optimal LC-MS urine peptidome analysis and represents an excellent replacement for the standard SPE method currently used. Since this report is based on the observation that was generated from a smaller sample set for the pure purpose of methodological evaluation, further application of the mSPE method on a larger sample cohort in clinical biomarker setting will provide the necessary clinical validation and utility.

## Methods

In this study we compared two peptide isolation methods to isolate urine peptides for peptidome analysis by LC-MS. First, we applied a centrifugal filtration method to isolate a native, small MW (<10 kDa) peptide fraction from urine following a protocol we developed for sample preparation for the proteomic analysis of urine [4]. The filtrate containing the peptide fraction was then subjected to two separate methods for peptide enrichment and purification. In the first method, we applied a hydrophilic-lipophilic-balanced reversed-phase sorbent-based solid phase extraction (SPE) method. SPE used hydrophilic-lipophilic-balanced reversed-phase sorbent-based (a mixture of two monomers, hydrophilic N-vinylpyrrolidone and lipophilic divinylbenzene) solid phase extraction. Whereas the second method called as modified SPE (mSPE) used peptides isolated from SPE and further purified the peptides with a processed silicon carbide resin in a pH dependent manner. The activation of the resin and peptide binding involved a low pH buffer (pH 3.5-4) and elution is performed with a Na-phosphate buffer (pH 12.5).

### Study subjects and samples, urine collection, initial processing, and storage

The study used a pool of urine samples obtained from 5 patients with biopsy confirmed [24] acute rejection of renal allografts (AR). Overall schematics of the method used in this study are summarized in Figure 1. Standard methods recommended by the Human Kidney and Urine Proteome Project (HKUPP) were followed during collection and processing of urine samples in the kidney transplant clinic. In our previous report we established protocols that allowed for stable urine collection from multicenter clinical studies [25], where delays in storage and processing can be encountered. With our protocols, urine samples can be safely stored up to 1 h at room temperature and up to 12 h at 4°C without significant protein degradation; samples do not require the addition of protease inhibitors to improve sample integrity if stored at either 4°C or −80°C within 72 h of collection; and centrifugal filtration was the optimal processing method. Second morning void, mid-stream urine samples (50–100 mL) were collected in sterile containers and centrifuged at 2000 × g for 20 min at room temperature within 1 h of collection. The supernatant was separated from the pellet containing any particulate matter including cells and cell debris. The supernatant was adjusted to pH 7.0 with 1 M Tris–HCl and stored at −80°C until further analysis. In order to ensure minimum impact of freeze-thaw cycles, we aliquotted urine samples into 10 mL aliquots (5–10 tubes per sample) prior to freezing, to ensure that multiple assays could be done without multiple freeze-thaw cycles. Our method utilizes 10 mL urine so each aliquot only needs to be thawed once for a single experiment. At the time of peptide extraction, urinary native peptides were separated from larger molecular mass intact proteins by filtering the supernatant through Amicon Ultra centrifugal filtration tubes (10 K molecular weight cutoff, Millipore, Bedford, MA). A 10 mL portion of urine was centrifuged for 20 min at 3000 × g at 10°C. The filtration unit was initially washed with 10 mL of deionized water to remove traces of glycerine by centrifuging the tube at 3000 × g for 10 min. This was followed by the addition of 10 mL of urine to the filter tube and centrifuged at 3000 × g for 20 min. The filtrate was transferred to a 15 mL Falcon tube and stored at −80°C until time for analysis.

### Sample preparation

A pool of 5 urine samples of 20 mL each, from patients with biopsy confirmed AR was assembled. The pooled urine sample was split into 10–2 mL aliquots. For this step we used 4–2 mL aliquots for the evaluation of the SPE method and 4–2 mL aliquots for the mSPE method that included the Proteospin column (Norgen Biotek, Thorold, Canada).

An Oasis HLB extraction cartridge (Waters, Milford, MA) was chosen for the initial peptide extraction. Two mL of 100% acetonitrile were passed through the column before equilibrating with 5 mL of 0.1% trifluoroacetic acid (TFA) at a rate of 1 drop/s. The pH of the peptide samples was adjusted to pH 3.0 with 50% TFA and the peptides were then loaded onto the cartridge. The cartridge was washed with 5 mL of 0.1% TFA followed by elution with 1.5 mL 0.1% TFA in 70% acetonitrile mixture. The elution step was repeated a second time and the volume of the eluted peptides were brought to 500 μL by SpeedVac concentration. The resulting peptide mixture was then extracted with 1 mL ethyl acetate by mixing and vortexing for 1 min and discarding the upper organic layer.

#### Solid phase extraction (SPE)

For the SPE extraction we repeated the previously described procedure using an Oasis HLB extraction cartridge for the initial peptide extraction. Two mL of 100% acetonitrile was passed through the column before equilibrating with 5 mL of 0.1% trifluoroacetic acid (TFA) at a rate of 1 drop/s. The peptide samples were adjusted to pH 3.0 with 50% TFA and the peptides were then loaded onto the cartridge. The cartridge was washed with 5 mL of 0.1% TFA followed by the elution step with 1.5 mL 0.1% TFA in 70% acetonitrile. The peptide extract was aliquoted and stored at −80°C until time for further analysis.

#### Enrichment of Low-MW endogenous peptides in Urine (using ProteoSpin™ Urine Protein Concentration Micro Kit Product-Product # 17400 (NOrgen Biotek, Thorold, Canada))(mSPE method)

For the mSPE method, an initial peptide extraction using an Oasis HLB extraction cartridge was executed, followed by ethyl acetate extraction method as mentioned previously. To the peptide extract after ethyl acetate extraction, we added 40 μL of pH Binding Buffer (pH 3.5) to 200 μL of urine peptide sample to adjust to pH 3.5. The provided spin column was assembled with a collection tube and 500 μL of Column Activation and Wash Buffer (both ph3.5) was added to the column. The column with processed silicon carbide was centrifuged for 2 min at 3300 × g. The activation step was repeated a second time and 500 μL of the pH-adjusted urine peptide extract from the Oasis HLB cartridge was loaded onto the column and centrifuged for 2 min. The peptide extract was passed through the column, 500 μL of Column Activation and Wash Buffer was added to the column, and the column was centrifuged for 2 min. The flow-through was discarded and the step was repeated one more time. Before the elution step 4.65 μL of Neutralizer was added to a fresh 1.7 mL Elution Tube and the column was transferred to the Elution Tube. 50 μL of Elution Buffer (10 mM sodium phosphate, pH 12.5) was added to the column and it was centrifuged for 2 min to elute bound peptides.

### LC-MS analysis

Peptide mixtures were analyzed on a high resolution, reversed-phase capillary LC system coupled with a Thermo Fisher Scientific LTQ-Orbitrap Velos MS (San Jose, CA). The automated LC system was custom built using two Agilent 1200 nanoflow pumps and one Agilent 1200 capillary pump (Agilent Technologies, Santa Clara, CA), and a PAL autosampler (Leap Technologies, Carrboro, NC). Full automation was made possible by custom software that allowed for parallel event coordination and, therefore, approximately 100% of the MS duty cycle, through the use of two trapping and analytical capillary columns, was achieved. Capillary reversed-phase columns were prepared in-house by slurry packing 3-μm Jupiter C18 (Phenomenex, Torrence, CA) into 35-cm × 360 μm o.d. × 75 μm i.d fused silica (Polymicro Technologies Inc., Phoenix, AZ). Trapping columns were prepared similarly, but using 3.6 μm Aeris Widepore XB-C18 packed into a 4 cm length of 150 μm i.d. fused silica. Mobile phases consisted of 0.1% formic acid in water (A) and 0.1% formic acid acetonitrile (B) operated at constant flow of 300 nL/min with a gradient profile over the course of 100 min as follows (min:%B); 0:5, 2:8, 20:12, 75:35, 97:60, 100:85. Sample injections (5 μL) were trapped and washed on the trapping columns at 1.5 μL/min for 20 min prior to alignment with analytical columns. Two-column operation also allowed for columns to be ‘washed’ (shortened gradients) and re-generated off-line without any cost to duty cycle.

MS analysis was performed on a LTQ-Orbitrap Velos mass spectrometer outfitted with a custom electrospray ionization (ESI) interface. Electrospray emitters were custom made by chemically etching 150 μm o.d. × 20 μm i.d. fused silica [25]. The heated capillary temperature and spray voltage were 350°C and 2.2 kV, respectively. Full MS spectra were recorded at a resolution of 100 K (for ions at m/z 400) over the range of m/z 400–2000 with an automated gain control (AGC) value of 1e6. MS/MS was performed in the data-dependent mode with an AGC target value of 3e4. The ten most abundant parent ions, excluding single charge states, were selected for MS/MS using high-energy collisional dissociation (HCD) with a normalized collision energy setting of 40%. A dynamic exclusion time of 45 s was used.

### Peptide and protein identifications

LC-MS/MS raw data were converted into “.dta” files using Extract_MSn (version 3.0) from Bioworks Cluster 3.2 (Thermo Scientific). MS/MS spectra were identified based on database searching against a human protein database (UniprotKB, released 2010–05) using both SEQUEST (version 27, revision 12) and MS-Align + algorithms [26]. MS-Align + is an algorithm designed for intact protein identification and is capable of identifying high mass peptides from MS/MS data. Filtering criteria using either MS-Generating Function Score (MS-GF) <1E-10 or MS-Align false discovery rate (FDR) <0.05 were applied to ensure the FDR of final peptide identifications were below 1% based on a decoy database searching strategy [27].

## Competing interests

The authors declare that they have no competing interests.

## Authors’ contributions

TS, CN, SH, HD and WQ contributed in experiment design and data collection. TS, WQ, DC, and MS contributed in study design, data analysis and manuscript preparation. All authors read and approved the final manuscript.

## Authors’ information

David G Camp and Minnie M Sarwal are joint senior authors.

## Supplementary Material

Additional file 1: Figure S1 (A) LC-MS chromatograms of two process replicates of the mSPE protocol; (B) LC-MS peak intensity correlation plot of two mSPE protocol replicates. Click here for file

Additional file 2 List of 1066 peptides identified in this study. Click here for file
