# Supplementary material for: Optimization for peptide sample preparation for urine peptidomics
Source: Clin Proteomics. 2014 Feb 25;11(1):7. doi: 10.1186/1559-0275-11-7 (PMC3944950; doi:10.1186/1559-0275-11-7)
Supplement: Additional file 1: Figure S1 — (A) LC-MS chromatograms of two process replicates of the mSPE protocol; (B) LC-MS peak intensity correlation plot of two mSPE protocol replicates. [file 1559-0275-11-7-S1.pdf]

Supplemental Figure 1

(A) (B)

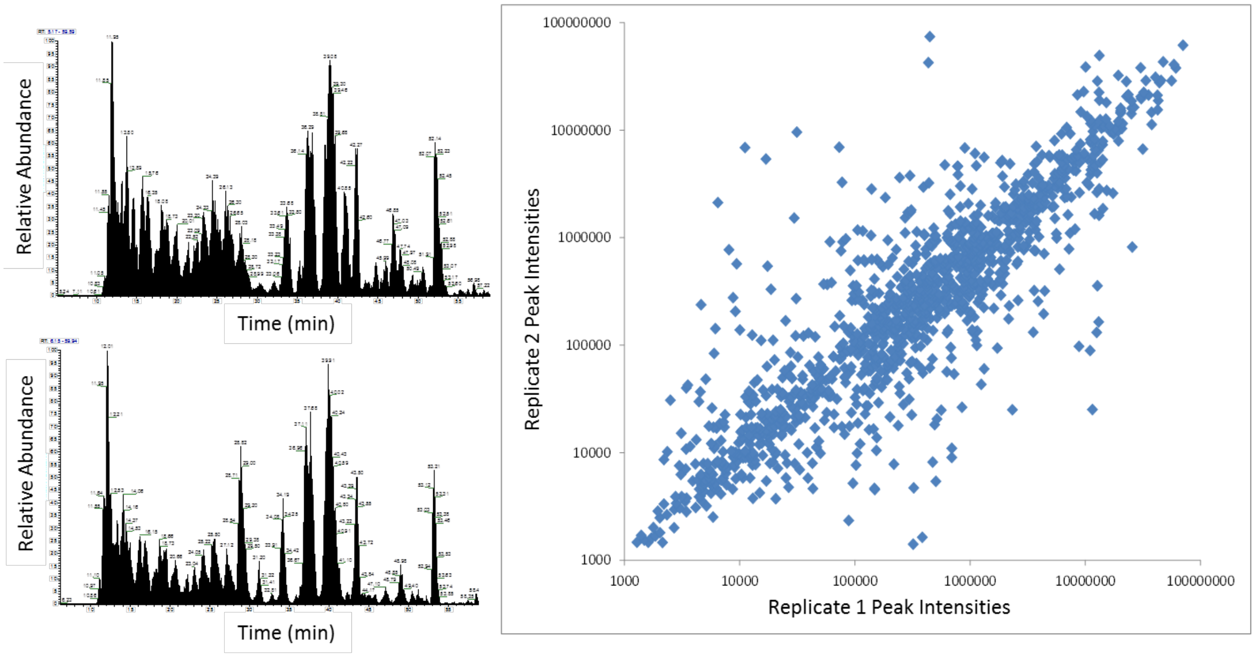

Supplemental Figure [Legend] (A) LC-MS chromatograms of two process replicates of the mSPE protocol; (B) LC-MS peak intensity correlation plot of two mSPE protocol replicates.
